# Mixed adenoneuroendocrine carcinoma of the gallbladder: a case report and literature review

**DOI:** 10.3389/fonc.2025.1584744

**Published:** 2025-07-31

**Authors:** Fan-Hua Kong, Guo-Dong Xu, Chang-Qing Liu, Hao Ma, Yun-Xuan Zou, Xiu-Feng Li

**Affiliations:** ^1^ Precision Pathological Diagnosis Center, Weifang People’s Hospital (First Affiliated Hospital of Shandong Second Medical University), Weifang, Shandong, China; ^2^ Department of Nuclear Medicine, Weifang People’s Hospital (First Affiliated Hospital of Shandong Second Medical University), Weifang, Shandong, China; ^3^ Department of Anesthesiology, Weifang People’s Hospital (First Affiliated Hospital of Shandong Second Medical University), Weifang, Shandong, China

**Keywords:** carcinoma, gallbladder, adenoneuroendocrine, adenocarcinoma, neuroendocrine carcinoma

## Abstract

Adenocarcinoma (AC) and neuroendocrine carcinoma (NEC) in the gallbladder originate from glandular epithelium and neuroendocrine cells, respectively. AC and NEC are called mixed AC-NEC (MANEC) when they occur in the same tumor. The present study presents the case of a 57-year-old female individual who was diagnosed with gallbladder space. Gallbladder, S4b+5 resection of liver and hepatic pedicle lymph -node dissection were performed. Postoperative pathology confirmed MANEC in the gallbladder. No metastasis was found in the liver and pedicle lymph nodes. Given the rarity and high degree of malignancy of MANEC originating in the gallbladder, the previous literature was reviewed to enhance the understanding of the diagnosis and treatment of this tumor.

## Introduction

Mixed neuroendocrine-non-neuroendocrine neoplasms (MiNENs) are a type of mixed epithelial tumor. The 2019 fifth edition of the World Health Organization (WHO) Classification of Digestive System tumors classifies tumors composed of neuroendocrine and non-neuroendocrine components as MiNENs, each of which is morphologically and immunohistochemically recognizable as a separate component, and constituting ≥30% of the tumor component. Mixed adenocarcinoma and neuroendocrine carcinoma (MANEC) is a specific subtype of MiNENs, which is more common in the stomach, colorectal and appendix, and is rare in the gallbladder (1). Based on the statistical results from Europe, the incidence of MiNENs is less than 0.01/100,000 cases per annum, and the common sites of origin of MiNENs are in descending order, the appendix (60.3%), colon-rectum (14.5%), and rarely biliary tract (1.6%), and two-thirds of cases in the biliary tract primarily arise from the gallbladder. MiNEN of the gallbladder is more common in women than men with a male-to-female ratio of 0.22 (2). While >1/3 of gallbladder NEC is associated with non-neuroendocrine components, the most common being AC (3). The present study reports the clinicopathological features of one case of MANEC in the gallbladder and reviews the literature to enhance the understanding of this neoplasm.

## Case report

Female, 57 years old, physical examination revealed a mass in the gallbladder. The chief complaint was no obvious discomfort. The patient has a history of hypertension for 4 years, with a maximum blood pressure of 180/90 mmHg. She is treated with oral irbesartan, and blood pressure is well-controlled. Underwent minimally invasive surgery for femoral head injury 14 years ago. Denies history of other diseases, infectious diseases such as hepatitis B or tuberculosis, or close contact with such infections. No family history of genetic disorders or similar conditions. The results of the laboratory tests were as follows: Alanine aminotransferase, 152 U/l (normal range, 0–40 U/l); aspartate aminotransferase, 88 U/l (normal range, 0–35 U/l), direct bilirubin 8.4 µmol/l (normal range, 0-8.0 µmol/l). Preoperative blood tests revealed a white blood cell count of 17.21×10^9^/L (normal range, 3.5-9.5 10^9^/L); absolute neutrophil count of 14.62×10^9^/L(normal range,1.8-6.3 10^9^/L), absolute lymphocyte count of 1.80×10^9^/L(normal range,1.1-3.2 10^9^/L), and platelet count of 335×10^9^/L(normal range,125-350 10^9^/L), with a neutrophil-to-lymphocyte ratio (NLR) ≈18 and platelet-to-lymphocyte ratio (PLR) ≈186.

**Figure 1:**
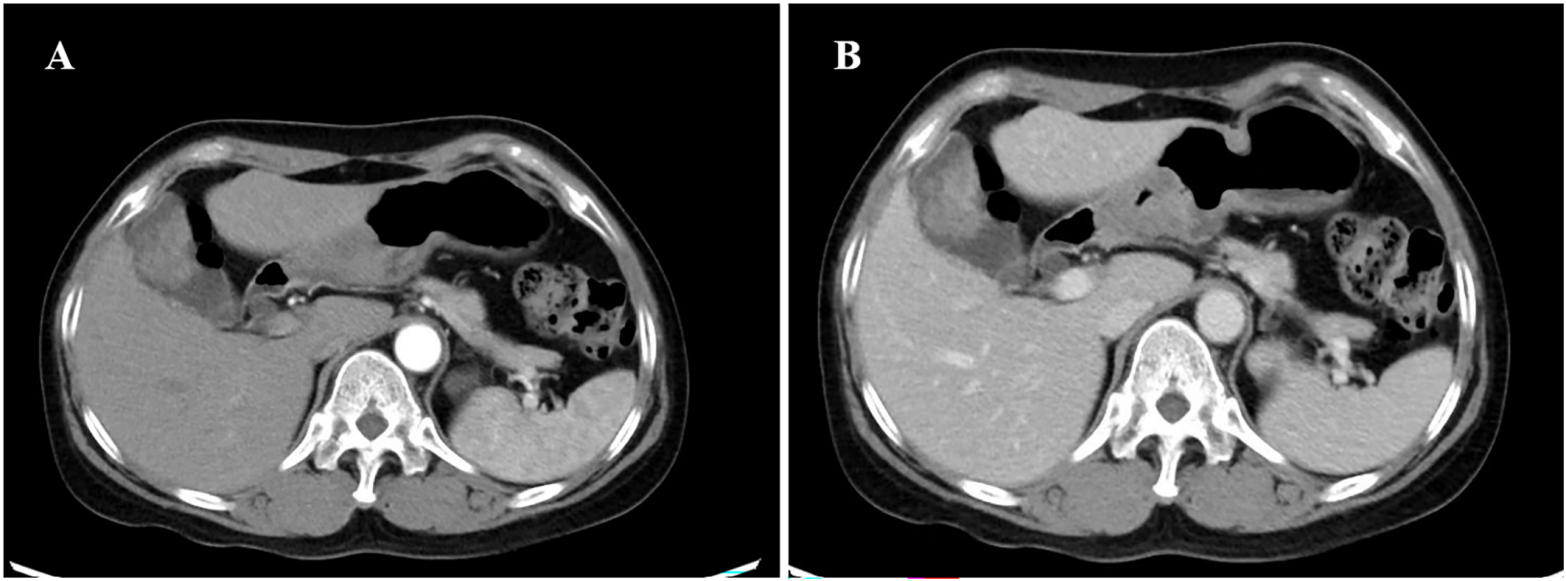
CT of the axial tomography of the abdomen and pelvis. CT scan showing a slightly high-density lesion **(A, B)** in the gallbladder, 3.8cm×2.6cm in size, with continuous and significant enhancement on enhanced scan. CT, computed tomography.

Computed tomography (CT) showed that the gallbladder was enlarged and irregular soft tissue density shadow was found on the inner wall, ranging from 3.8 cm x 2.6 cm. Enhancement was observed after enhancement (**Figures 1A, B**), and the boundary between gallbladder and liver was unclear. Chest CT and breast ultrasound showed no space-occupying lesions. After contraindications were excluded, gallbladder, S4b+5 section resection of the liver and hepatic pedicle lymph node dissection were performed.

Surgical specimens were fully fixed with 10% neutral formalin, dehydrated and made transparent using an automatic dewatering machine, followed by conventional paraffin embedding, sectioning, and hematoxylin-eosin (HE) staining. At the same time, appropriate tumor tissue wax blocks were selected and immunohistochemical staining was performed using En Vision method. All the reagents used for immunohistochemistry were purchased from Beijing Zhongshan Jinqiao Biotechnology Co., Ltd (ZSGB-BIO). The main antibodies used for immunohistochemistry (IHC). Epithelial immunohistochemical markers: mouse anti-CK (ZM-0069; ZSGB-BIO) and rabbit anti-CK7 (ZA-0573; ZSGB-BIO), intestinal-derived epithelial markers: rabbit anti-CDX-2 (ZA0520; ZSGB-BIO), neuroendocrine markers: mouse anti-CD56 (ZM-0057; ZSGB-BIO), rabbit anti-Syn (ZA-0506; ZSGB-BIO), mouse anti-CgA (ZM-0076; ZSGB-BIO) and rabbit anti-CD117 (ZA-0523; ZSGB-BIO), Somatostatin receptor marker: rabbit anti-SSTR-2 (ZA-0587; ZSGB-BIO), Tumor suppressor gene: mouse anti-p53(ZM-0408; ZSGB-BIO), Cell proliferation index marker: mouse anti-Ki-67 (ZM-0166; ZSGB-BIO). The experimental procedures were carried out in strict accordance with the kit instructions.

### Microscopic observation

**Figure 2:**
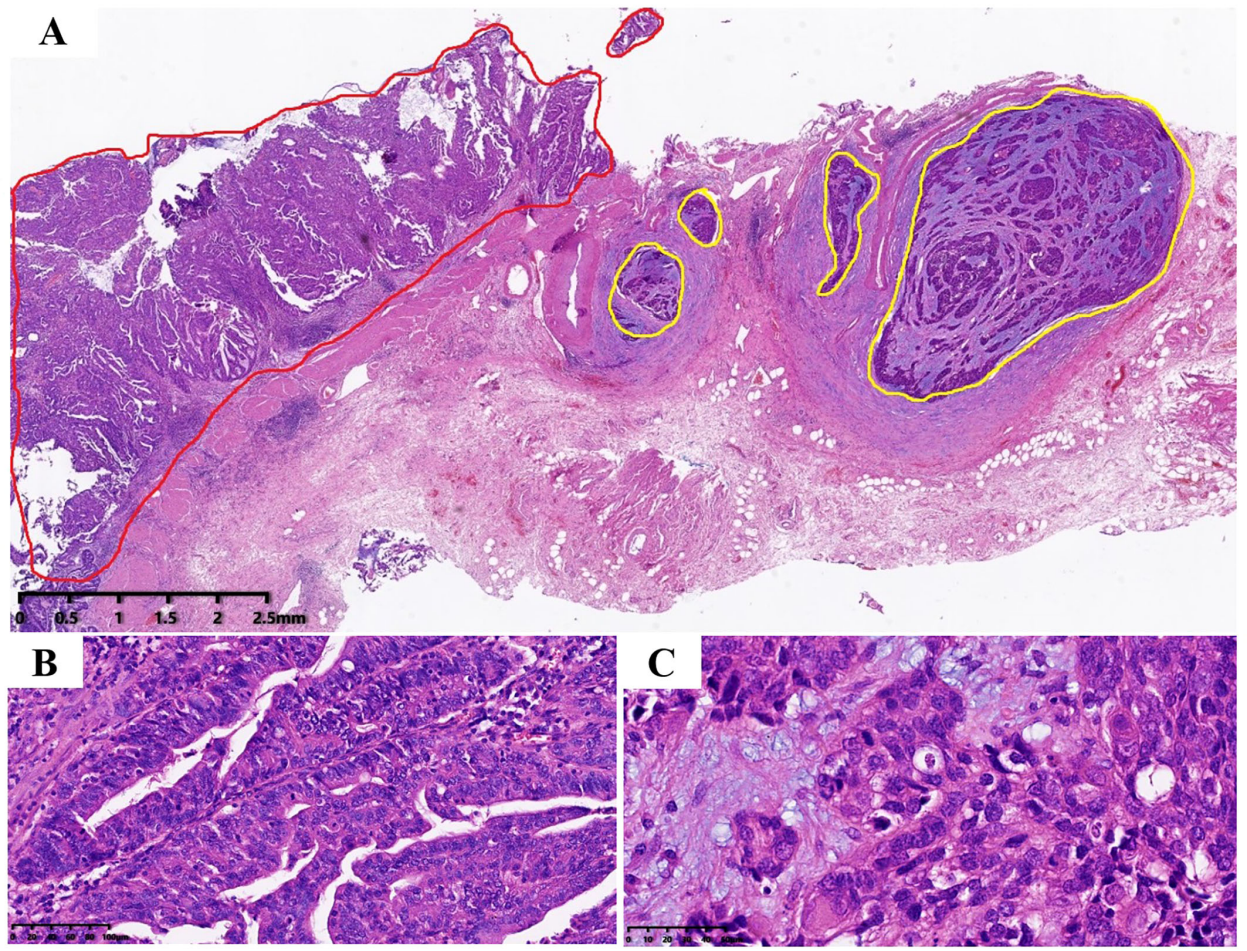
Tumor hematoxylin and eosin staining. **(A)** Tumor consists of two components, the red area is AC; while the yellow area is neuroendocrine carcinoma, which is located on the deep side of the tumor (magnification, ×10), which infiltrates the whole layer of the gallbladder wall; **(B)** The AC component, which is arranged adenoid (magnification, ×200). **(C)** The neuroendocrine carcinoma, with nest flakiness distribution, high nucleo-plasma ratio, fine granular chromatin, easy-to-see mitotic images and focal necrosis (magnification, ×400).

The tumor invaded the entire wall of the gallbladder, and the tumor was composed of two completely independent components (**Figure 2A**). AC components were found in adenoid and tubular arrangement (**Figure 2B**), and neuroendocrine carcinoma components were found in nests and solid lamellar arrangement. The mitotic image was evident (**Figure 2C**).

**Figure 3:**
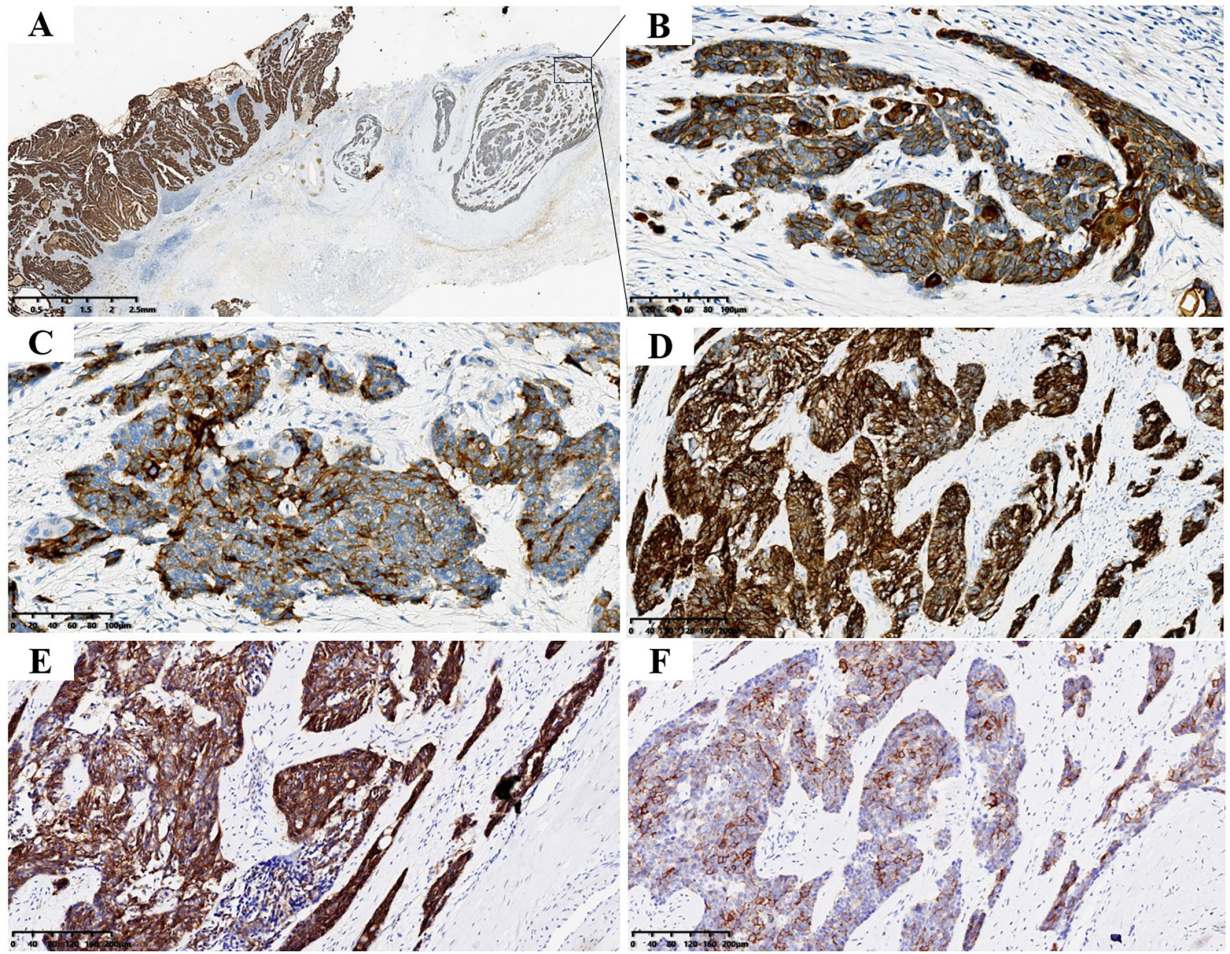
Tumor immunohistochemical staining. **(A)** Both AC and NEC expressed CK (magnification, ×10and ×200). **(B)** B is enlarged view of the portion shown by the arrow in **(A, C)** NEC expressed Syn (magnification, ×200). **(D)** NEC expressed CD56 (magnification, ×200). **(E)** NEC expressed SSTR-2 (magnification, ×200). **(F)** NEC expressed CD117 (magnification, ×200).

Immunohistochemical results showed that both tumor components expressed human pan-cytokeratin (CK; **Figures 3A, B**) and scattered expressed recombinant transcription factor 2 (CDX2). Cell keratin 7 (CK7), CK19 and cell proliferation antigen index (Ki-67 index; 50%) were positively expressed in AC components. AC components did not express neural cell adhesion molecules (CD56), synaptophysin (Syn), chromogranin A (CgA), and somatostatin receptor-2 (SSTR-2), stem cell growth factor receptor (CD117); NEC component positively expressed Syn (**Figure 3C**), CD56 (**Figure 3D**), and SSTR-2 (**Figure 3E**), CD117 (**Figure 3F**). A small proportion of NEC components expressed CgA and did not express CK7 and CK19. The index of Ki67 in NEC component was 75% (**Supplementary Figure 1A**), Neither AC nor NEC component expressed P53, indicating that the P53 gene was mutant in tumor (**Supplementary Figure 1B**).

### Pathological diagnosis

Gallbladder combined with immunohistochemistry was consistent with MANEC, in which AC (medium-low differentiation) accounted for 65% and NEC accounted for 35%, invading the whole layer of the gallbladder wall without involving the serous membrane; no metastases were found in liver tissue and pedicle lymph nodes.

### Treatment and follow-up

The patient did not receive radiotherapy or chemotherapy after surgery, and no recurrence or metastasis occurred within 18 months of follow-up.

## Literature review and discussion

MiNENs is a special type of mixed tumor, in which both neuroendocrine and non-neuroendocrine tumor components are present within the same tumor. If it is a mixture of AC and NEC, it is called mixed AC according to the 2010 WHO classification of digestive tumors. Therefore, the present authors searched the English literature on mixed adenoneuroendocrine carcinoma of gallbladder from 2010 to present in PUBMED and summarized the clinical features and immunohistochemical tests of the cases involved in the literature and the present case (**Table 1**). At the same time, the clinical features, pathological features, tissue origin, genetic alteration, differential diagnosis and treatment prognosis of MANEC in the gallbladder were discussed.

**Table 1:** Clinicopathological data of 22 literature cases of mixed adenocarcinoma neuroendocrine carcinoma of the gallbladder.

NO	Year^RF^	Age/sex	Clinical presentation	Depth of infiltration	Lymph node metastasis	NEC							Surgery and/or chemotherapy	Outcome (Survival\ Death)
						Components	Antibody							
							Syn	CgA	CD117	CDX-2	P53	SSTR-2		
1	**2010 (** 6)	68/F	Jaundice	hif	+	LCNEC	+	+	N☆	N	+	-	Cholecystectomy with regional lymph node dissection	RF (12)
2	**2010 (** 10)	48/F	History of primary liver cancer	T3	-	NEC	+	+	N	N	N	N	Cholecystectomy and hepatobidectomy	NM
3	**2011 (** 7)	48/F	Right upper abdominal discomfort /lump	T3	+	SCNEC	-*	-	N	-	N	N	Extended radical cholecystectomy and chemotherapy	RF (18)
4	**2011 (** 8)	48/F	Abdominal pain	hif	+	LCNEC	N*	+	+	N	N	N	Cholecystectomy and hepatobidectomy	NM
5	**2012 (** 3)	70/F	Cholecystolithiasis	hif	+	SCNEC	+	+	+	N	N	-	NM	NM
6		70/F	NA	se	-	LCNEC	+	+	+	N	N	-	NM	NM
7		60/F	Cholecystolithiasis	ss	+	SCNEC	+	+	+	N	N	-	NM	NM
8		50/F	NA	hif	+	LCNEC	+	+	+	N	N	+	NM	NM
9	**2012 (** 9)	55/F	Abdominal pain	T3	-	SCNEC	+	+	N	N	N	N★	Chemotherapy Radical cholecystectomy	RF (7)
10	**2012 (** 11)	72/F	Mass on the right rib	T2b	N2	SCNEC	+	+	N	N	N	N	Chemotherapy, anatomy	D (2)
11	**2013 (** 12)	45/M	Abdominal pain	T3	+	LCNEC	+	+	N	N	N	N	Cholecystectomy chemotherapy	NM
12	**2014 (** 13)	34/F	Abdominal pain	T3	+	NEC	+	+	N	N	N	N	Cholecystectomy plus hepatobed resection and subhepatic resection	S (4)
13	**2015 (** 14)	63/F	Abdominal pain	T2a	-	LCNEC	+	+	N	N	N	N	cholecystectomy	RF(12)
14	**2015 (** 15)	55/F	Postprandial RuQ pain	SE	+	LCNEC	+	+	N	+	-	N	cholecystectomy	NM
15	**2015 (** 16)	65/F	NA	T3	-	NEC	+	+	N	N	N	N	Biopsy	S(2)
16	**2015 (** 17)	62/F	Asymptomatic	SE	-	NEC	+	N	N	N	N	N	cholecystectomy	RF(24)
17	**2018 (** 18)	43/F	RUQ pain	T3	-	SCNEC	+	+	N	N	N	N★	Cholecystectomy, chemoradiation	S(21)
18	**2019 (** 19)	74/F	Abdominal pain fever	SE	-	LCNEC	+	+	N☆	N	+	N	cholecystectomy	S(7)
19	**2022 (** 2)	56/F	Abdominal pain Weight loss	T4	+	SCNEC	+	N	N☆	N	N	N★	Cholecystectomy plus hepatic segmentectomy plus concurrent chemoradiotherapy	S(13)
20	**2022 (** 2)	70/F	Abdominal pain	mp	+	LCNEC	strong+	weak+	-	-	stong+		cholecystectomy	D(30)
21		64/F	Abdominal pain nausea vomit	ss	+	LCNEC	strong+	stong+	-	-	-		Cholecystectomy and hepatectomy	S(11)
22	Present	57/F	Asymptomatic	T3	-	SCNEC	strong+	little +	+	little+	-	+	cholecystectomy with the cleaning of the regional lymph nodes plus segmental liver resection	S(12)

M, Male; F, Female; NM, Not mentioned; SCNEC, Small cell neuroendocrine carcinoma; LCNEC, Large cell neuroendocrine carcinoma; Mp, Muscle propria; se The tumor invades the connective tissue around the muscle of the organ No serous membranes were involved, ss Subserosal invasionse: tumor penetrated the serosa without invasion of adjacent structures, hinf Hepatic infiltration; cholecystectomy with RL: cholecystectomy with the cleaning of the regional lymph nodes,cholecystectomy with L: cholecystectomy with segmental liver resection, N* Not done,However, CD56 was expressed positively,-* Negative expression, However, CD56 was expressed positively. N☆ Not done,However,Prostem-cell hypothesis, N★ Not done,However,somatostatin treatment is effective, RF recurrence-free.

Bolded numbers represent the year in which each case was published.

### Clinical features

The clinical features of MANEC are non-specific (4), and the clinical symptoms of MANEC occurring in the gallbladder are similar to those of other types of gallbladder cancer. Mouyed Alawad et al. summarized 30 cases of similar gallbladder lesions, 90% of which were accompanied by different degrees of abdominal pain, abdominal discomfort, gallstone, etc. (5). Among the 22 patients with gallbladder MANEC collected in that study, 90% of them had symptoms such as abdominal pain, nausea and vomiting of different degrees (**Table 1**). Ren et al. summarized 74 cases of gallbladder MiNENs with a median age of 59 years (2). There was no significant difference in gender and 3/4 of the patients were found to be in the advanced stage [(American Joint Committee on Cancer, AJCC (eighth edition stage T2-T4)], 58% were accompanied by local lymph node metastasis or/and adjacent liver invasion, liver metastasis, umbilical or distant bone metastasis. Lymph node metastasis was present at the time of discovery in 59% of the cases that were counted (**Table 1**).

Therefore, these diseases have no special clinical manifestations and are easy to be confused with other gallbladder diseases (such as cholecystitis, gallstones, gallbladder polyps, etc.). Other methods, such as imaging and pathology, are often needed to make a definitive diagnosis. According to the overexpression of SSTR on the cell surface of most Neuroendocrine tumors (NETs), radionuclide-labeled somatostatin analogs can bind closely to SSTR, positron emission tomography (PET) is used to detect the presence of NETs by detecting receptor-dependent metabolic changes. Previous studies showed that SSTR imaging is more suitable for the diagnosis of NET (G1, G2 and G3), while NEC is not recommended because of its low positive rate and degree of SSTR expression. PET-CT was more suitable for 18F-fluorodeoxy glucose (18F-FDG) (20). Because the imaging examination can only identify the single tissue component of the tumor, the diagnostic accuracy of this disease is low, which is not conducive to the diagnosis and later treatment of the disease (21), and also highlights the important role of histopathology in the diagnosis of this type of disease (22).

### Pathological features

In most gallbladder MANEC, AC components are usually tubular or papillary, with varying degrees of differentiation, mainly located on the tumor surface, and may be accompanied by deep tissue infiltration. Neuroendocrine carcinoma tissues may originate from the deep mucosa of the gallbladder and infiltrate the gallbladder wall deeper. In the present case, AC was located on the surface of the tumor, infiltrating the whole lamina propria, while neuroendocrine carcinoma was in the muscular layer and subserous layer, without involving the mucosal layer. At the same time, it was found that the type of NEC in MANEC could be small or large cell carcinoma, which accounted for 44.4% and 55.6% of the statistical cases, respectively (**Table 1**).

The two tumor components in MANEC express different immunohistochemical markers. It was reported that CgA and Syn markers are highly sensitive to the diagnosis of MANEC, and up to 92.3% of NEC components express CgA and/or Syn (4). Among the cases collected in this study, 90.9% were found to express Syn and CgA to varying degrees, while only 0.48% expressed CD56 (**Table 1**).

### Tissue origin

The origin of MiNENs is still unclear, but it may have originated from common stem cells that eventually differentiated into two different types. The normal mucous membrane of the gallbladder does not contain neuroendocrine cells except in the neck area. There are three hypotheses about the origin of neuroendocrine carcinoma in primary gallbladder MANEC; i) The mucosa of gallbladder may undergo gastric mucosa or intestinal mucosa metaplasia under the stimulation of chronic inflammation and the neuroendocrine cells contained in this metaplasia mucosa may be a source of neuroendocrine tumors; ii) neuroendocrine tumors can be generated by endodermal stem cells or progenitor cells with multidirectional differentiation potential (8, 23, 24); iii) AC may abnormally differentiate into neuroendocrine cells through a ‘neoplasia’ process to form neuroendocrine tumors in MANEC. Acosta et al. mentioned that CDX2 is a marker of intestinal metaplasia (15). c-kit (CD117) can be used as a marker of stem cell function (25). Only five cases were tested for CDX-2 and one of them was positive, which supported the metaplasia hypothesis. The other eight cases were tested for CD117, of which six were positive, and the other three were not confirmed by immunohistochemistry but supported the stem cell hypothesis.

### Genetic changes

Emerging genomic data prove that NETs and NEC are unrelated. Specifically in the pancreas, multiple endocrine neoplasm type 1 (MEN1), death-associated protein 6, and alpha thalassemia/mental Frequent mutation of retardation (ATRX) is diagnostic for highly differentiated NETs, while there are no mutations in poorly differentiated NEC, which conversely has mutations in TP53, retinoblastoma protein 1 (RB1), and other cancer-associated genes (1). TP53 mutations can be detected by immunohistochemical staining of p53 protein products or somatic mutational profiles, etc. In gallbladder MANEC, higher p53 mutations are shown, which may be associated with shorter survival (26). In this study, six of twenty-two cases of MANEC detected P53 showed mutation and four of them were accompanied by lymph node metastasis, suggesting poor prognosis.

### Differential diagnosis

Gallbladder MANEC usually needs to be differentiated from the following diseases; i). The composition of NEC and poorly differentiated AC overlap in tumor cell morphology and structure, which can be distinguished by immunohistochemistry. Poorly differentiated AC does not express neuroendocrine markers, such as Syn and CgA; ii) both NEC components and NETs express neuroendocrine markers, and the key points of differentiation are based on the degree of tissue differentiation and cell proliferation activity. However, for patients with difficulty in differentiating NEC from NETG3, deletion of ATRX, MEN1 or p27 and/or positive staining of SSTR-2 or SSTR5 were more supportive of NETG3 diagnosis. Conversely, absence of expression of RB1, diffuse or complete absence of P53, and absence of expression of SSTR-2 or SSTR-5 supported the diagnosis of NEC (27). In addition, a small number of NETs may present with abnormal expression of P53 (28); iii). It is necessary to distinguish from metastatic poorly differentiated AC or NEC. At this time, a close medical history should be consulted. Metastatic tumors are often multiple and imaging examinations such as PET-CT are helpful for differential diagnosis.

### Treatment and prognosis

Therefore, the present is rare and there is no optimal treatment plan and consensus at present. After consulting relevant literature, surgical resection is the first choice. For early gallbladder cancer, such as stage T1N0, simple cholecystectomy is recommended (9). Extended radical cholecystectomy (29), including hepatic segment (IV and V) and lymph node dissection, is suitable for advanced gallbladder cancer (refer to AJCC 8th edition stage T2-T4 for pathological staging) and local hepatic invasion. Radical cholecystectomy may improve 5-year overall survival (9). Recent studies showed that somatostatin analogs such as SOM230(pasireotide) and octreode are the best treatment options for neuroendocrine tumors, with anti-proliferation, anti-angiogenesis and pro-apoptotic effects, thus achieving antitumor effects (30, 31). Therefore, somatostatin analog therapy is considered a new therapeutic approach, especially in cases where the somatostatin receptor is expressed on the surface of tumor cells. Among the twenty-two cases collected in this paper, only six cases were tested for SSTR, of which two cases showed positive SSTR and the other three cases showed effective somatostatin treatment although the index was not detected (18). NCCN (National Comprehensive Cancer Network) guidelines indicate that patients with unresectable neuroendocrine tumors can be treated with palliative surgery, adjuvant chemotherapy (platinum drugs), and somatostatin analogs (32). Wu et al. reported that neoadjuvant chemotherapy combined with somatostatin treatment transformed an unresectable MiNEN case into a case that could be radically resected, thus making radical surgery possible (9). In addition, it was reported in the literature that postoperative adjuvant chemoradiation and somatostatin therapy have a good prognosis, indicating that chemotherapy combined with somatostatin analogs may have a good effect on the long-term prognosis of MiNEN patients (4).

Giannetta E et al. mentioned Pro-inflammatory signals promote tumorigenesis and development, and a link exists between chronic inflammation and the development of neuroendocrine neoplasms (NENs). It has been confirmed that hematological parameters and their derived ratios (neutrophil-to-lymphocyte ratio [NLR], platelet-to-lymphocyte ratio [PLR], lymphocyte-to-white blood cell ratio [LWR], systemic immune inflammation index [SII]) in patients can reflect the degree of systemic inflammation and are defined as novel prognostic and predictive biomarkers. High NLR and PLR are associated with shorter progression-free survival (PFS) (p=0.0001), but the definition of their thresholds still lacks consensus (33). None of the cases in Table mentioned inflammatory markers in serum. This patient had a preoperative NLR≈18 and PLR≈186. No recurrence has occurred during follow-up to date. The clinical value of inflammatory markers such as NLR, PLR, and pro-inflammatory cytokines in the diagnostic process, therapeutic efficacy assessment, prognosis evaluation, and long-term follow-up of NENs still requires further validation through more prospective studies.

In conclusion, the primary MAC-NEC of the gallbladder reported in this article is a rare and poorly differentiated malignant tumor. It shows no specific clinical symptoms, age of onset or gender, and is easily confused with other gallbladder diseases. Imaging examinations have certain diagnostic value, and histopathological diagnosis is the gold standard for definite diagnosis. However, there is no consensus on its tissue origin at present. The immunohistochemical results of this case support the stem cell hypothesis of origin. In terms of treatment, the extended cholecystectomy of liver tissue was chosen for this case, which is the same as some of the cases reviewed previously. Some of the latter patients chose chemotherapy and other drug treatments. Regarding prognosis, although the patient in this case has no discomfort at present, the absence of P53 gene expression in this case suggests a poor prognosis. In addition, most of the current reports on this type of disease are case reports, and more samples need to be collected to summarize and analyze its tissue origin, diagnosis, treatment and prognosis to enhance the understanding of this type of disease.

## Data Availability

The original contributions presented in the study are included in the article/Supplementary Material, further inquiries can be directed to the corresponding author/s.

## Supplementary material

Supplementary Figure 1 Tumor immunohistochemical staining. **(A)** Both AC and NEC do not expressed P53 (magnification,×40). **(B)** Both AC and NEC highly expressed Ki-67 (magnification,×40).
