# Supplementary figures and images for: Mixed adenoneuroendocrine carcinoma of the gallbladder: a case report and literature review
Source: Front Oncol. 2025 Jul 31;15:1584744. doi: 10.3389/fonc.2025.1584744 (PMC12350106; doi:10.3389/fonc.2025.1584744)

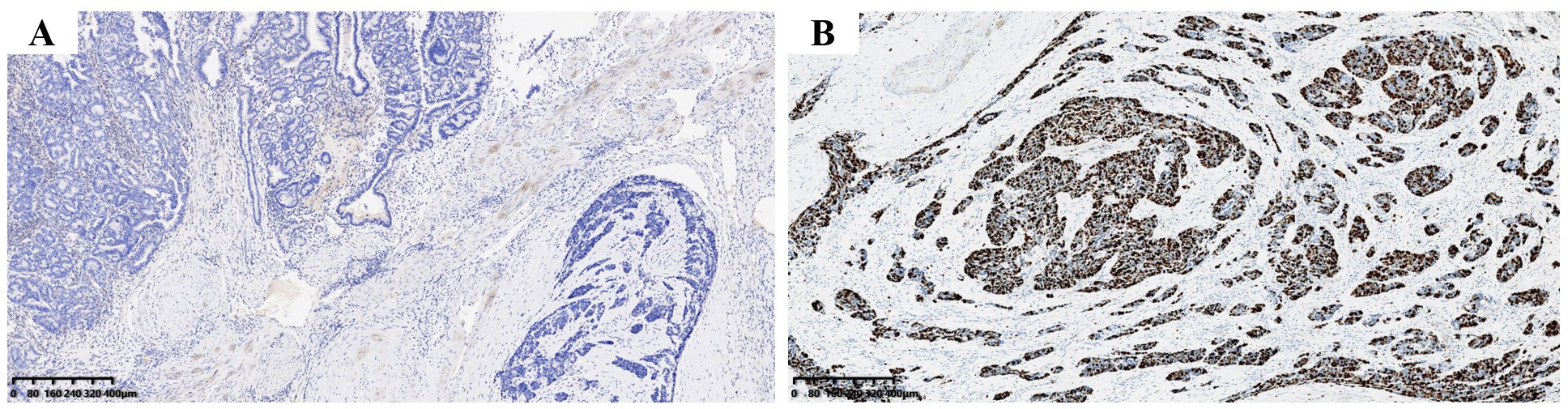

Supplement: Supplementary Figure 1 — Tumor immunohistochemical staining. (A) Both AC and NEC do not expressed P53 (magnification,×40). (B) Both AC and NEC highly expressed Ki-67 (magnification,×40). [file Image1.tif]
